# Social bonds affect anti-predator behaviour in a tolerant species of macaque, *Macaca nigra*

**DOI:** 10.1098/rspb.2012.1470

**Published:** 2012-08-01

**Authors:** Jérôme Micheletta, Bridget M. Waller, Maria R. Panggur, Christof Neumann, Julie Duboscq, Muhammad Agil, Antje Engelhardt

**Affiliations:** 1 Department of Psychology, Centre for Comparative and Evolutionary Psychology, University of Portsmouth, Portsmouth, UK; 2 Reproductive Biology Unit, Junior Research Group of Primate Sexual Selection, German Primate Centre, Göttingen, Germany; 3 Faculty of Mathematics and Natural Sciences, Bogor Agricultural University, Bogor, West Java, Indonesia; 4 Courant Research Centre ‘Evolution of Social Behaviour’, Georg-August-Universität Göttingen, Göttingen, Germany; 5 Department of Primatology, Junior Research Group of Primate Kin Selection, Max Planck Institute for Evolutionary Anthropology, Leipzig, Germany; 6 Institute of Biology, Faculty of Bioscience, Pharmacy and Psychology, University of Leipzig, Leipzig, Germany; 7 Département Ecologie, Centre National de la Recherche Scientifique, Physiologie et Ethologie, Strasbourg, France

**Keywords:** anti-predator behaviour, primate communication, social bond, cooperation, alarm calls, relationship quality

## Abstract

Enduring positive social bonds between individuals are crucial for humans' health and well being. Similar bonds can be found in a wide range of taxa, revealing the evolutionary origins of humans' social bonds. Evidence suggests that these strong social bonds can function to buffer the negative effects of living in groups, but it is not known whether they also function to minimize predation risk. Here, we show that crested macaques (*Macaca nigra*) react more strongly to playbacks of recruitment alarm calls (i.e. calls signalling the presence of a predator and eliciting cooperative mobbing behaviour) if they were produced by an individual with whom they share a strong social bond. Dominance relationships between caller and listener had no effect on the reaction of the listener. Thus, strong social bonds may improve the coordination and efficiency of cooperative defence against predators, and therefore increase chances of survival. This result broadens our understanding of the evolution and function of social bonds by highlighting their importance in the anti-predator context.

## 1. Introduction

The existence of strong enduring social bonds between individuals is a central feature of human societies. The quality and quantity of social relationships can improve mental health and reduce morbidity and mortality [1–3]. Studies in a wide range of taxa, from mice to non-human primates, show that these bonds are not uniquely human, which allows us to investigate the evolution and function of such strong positive relationships [4,5]. Typically, data from non-human animals suggest that social bonds provide fitness benefits in the within-group social context, but whether they also provides advantages in immediate survival contexts such as defence against predators, remains unknown [4]. Therefore, the aim of this study was to investigate the role that social bonds may have in the context of anti-predator behaviour using field playback experiments.

Strong positive social bonds often involve short- and long-term cooperative acts, providing individuals with substantial fitness benefits in the form of increased reproductive success and longevity [4,6]. For example, close affiliates reconcile more frequently than non-affiliates that facilitate the reparation and maintenance of valuable relationships [7–9]. In Tonkean macaques (*Macaca tonkeana*), a species characterized by tolerant social relationship, close social bond minimizes social instability and reduces stress by promoting affiliation between bystanders after conflicts [10]. Strong social bonds also enhance long-term grooming equity [11] and in olive baboons (*Papio hamadryas anubis*), females form special bonds with certain males of their group from which they receive assistance during conflict and thus protection from non-lethal harassment by group-members [12]. The role of social bonds in increasing offspring survival, either as a result of greater social integration [13,14], or increased protection against infanticide [15], is also well documented. In the context of communication, social bonds can influence patterns of vocal exchange used to maintain social relationships and/or to regulate spatial distances between individuals [16–18]. Social bonds can also facilitate the acquisition of valuable information in the social and ecological domain [19,20].

While it is clear that social bonds are extremely important in the within-group social context, their impact (if any) in the context of anti-predator behaviour has not been investigated [4]. Many animals emit specific vocalizations (i.e. alarm calls) when they detect predators that can lead to increased vigilance and/or flight from listeners, or to the gathering of group-members around the predator, often leading to cooperative attacks or harassment of the predator (i.e. mobbing [21,22], see electronic supplementary material, video S1). A specific class of alarm calls labelled ‘recruitment alarm calls’ leads other individuals to approach the caller and seems, at least to some extent, to enhance the coordination and the efficiency of cooperative defence against predators [21,23].

Just as strong affiliates preferentially assist each other during conflicts [12,24], they might be more responsive to each others' recruitment alarm calls, resulting in improved cooperative efforts against predators and reduced risks of being predated. At the same time, the trade-off between benefits and risks of predator defence will influence an individual's decision on whether to join in or not [25]. Predation can thus be expected to have produced important selective pressures on social relationships, in this way shaping the evolution of sociality [26]. Achieving a better understanding of the impact of social relationship quality on anti-predator behaviour will therefore help us to understand fully the ultimate functions underlying the formation of close enduring social bonds. This, however, is not an easy task: measuring the costs and benefits of sociality in relation to predation has proved to be challenging. Indeed, direct observation of predation is difficult, and so is the assessment of the fitness costs and benefits associated with anti-predator behaviour [27,28].

One way of circumventing these issues is to study alarm calling behaviour. This approach has deepened our understanding of how different strategies are used to counterbalance the costs associated with predation, and provided insight into the importance of relationship quality and context, even in urgent situations such predator encounter [22]. The acoustic structure of alarm calls can encode the identity of the caller and sometimes differs between age–sex categories, so listeners can assess the reliability of the caller and adapt their response accordingly [22,29,30]. In some primates and sciuridae, young individuals are more likely to produce alarm calls in non-threatening situations, and the responses to these calls differ from those given to calls produced by adults [31–34]. Social status and kinship also seem to influence alarm calling behaviour both in terms of vocal production and perception. High-ranking vervet monkeys (*Cercopithecus aethiops*) were more likely to engage in alarm calling behaviour than those low-ranking when a predator was encountered, and females alarm-called more often when in the presence of their kin compared with an unrelated juvenile [35]. In rhesus macaques (*Macaca mulatta*), playbacks of high-ranking individuals' alarm calls elicited stronger responses from conspecifics than calls from low-ranking individuals, and this pattern persisted even after repeated exposure to the stimuli [36]. Such findings clearly suggest that the nature and quality of the social relationship between callers and listeners as well as the context in which the alarm calls are uttered can be highly significant.

In the present study, we investigate whether and how the strength of the social bond between caller and listener influences anti-predator behaviour. By using field playback experiments, we test the hypothesis that crested macaques would increase their response to alarm calls produced by strong affiliates compared with individuals with whom they do not share a strong affinitive bond. Crested macaques are semi-terrestrial primates that live in multimale, multifemale groups. Compared with other macaque species, crested macaques are highly socially tolerant: social bonds usually have more weight than dominance or kinship on their social life [19,37,38]. In the wild, they encounter several potential predators, such as reticulated pythons (*Python reticulatus*), dogs (*Canis familiaris*) and humans [39,40] (J. Micheletta & J. Duboscq 2008–2011, unpublished data). When detecting a predator, all individuals normally produce series of alarm calls. When the calls are produced in reaction to the presence of a python, they lead other individuals to approach and engage in mobbing behaviour (see electronic supplementary material, video S1). Python-related alarm calls therefore seem to function as recruitment alarm calls [41].

To test the role played by social bonds during anti-predator behaviour, we first presented an artificial predator to obtain high-quality alarm calls. Then, we carried out acoustic analysis to determine whether the identity of the alarm caller could be discriminated by listeners. Finally, we played back the python-related alarm calls to the same adult females in two different conditions: when the call was produced by a close affiliate (affiliate condition) and when the call was produced by an individual with whom the subject does not share a strong affinitive bond (non-affiliate condition). Because of their high level of social tolerance, and social bonds being important in cooperative interactions, we expected that crested macaques would increase their reaction to recruitment alarm calls produced by strong affiliates.

## 2. Material and methods

### (a) Study site and subjects

This study is part of the Macaca Nigra Project, whose members investigate the biology of crested macaques living in the Tangkoko nature reserve, North Sulawesi, Indonesia, since 2006. A detailed description of the macaques' habitat can be found elsewhere [42,43]. We studied two groups of crested macaques (group R1 and group PB) between September 2010 and April 2011. The two groups comprised 60 and 80 individuals in total, with 20–22 and 24–26 identifiable adult females, respectively.

### (b) Assessment of the strength of social bonds

To estimate the strength of the social bond between two individuals, we used a composite sociality index (CSI; [44]) based on the frequencies of grooming interactions and close proximity (i.e. sitting within 1 m). These data were collected between April 2008 and April 2011 reaching a total of 60.4 ± 4.4 h of focal observation per female (range: 50.1–69.3 h per female). The CSI was calculated using the following equation:
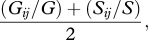
where *G_ij_* is the frequency of grooming given and received by members of the dyad *i,j*; *G* is the mean frequency of grooming for all dyads in the group; *S_ij_* is the frequency of sitting within 1 m for the dyad *i,j* and *S* is the mean frequency of sitting in contact for all dyads in the group. This composite index results in a score representing the extent to which a particular dyad deviates from the average of all dyads. Therefore, it characterizes the strength of the positive relationship for each dyad. Dyads with a high CSI have stronger bonds than the average dyad, whereas dyads with a low CSI have a weaker bond than the average dyad. We defined strongly bonded individuals (hereafter referred to as ‘affiliates’) as individuals sharing a CSI score greater than 1 s.d. above the mean of the group, and weakly bonded individuals (hereafter ‘non-affiliates’) as individuals having a CSI score lower than 1 s.d. below the mean of the group.

### (c) Model presentation and recording of alarm calls

To discriminate alarm calls from affiliates and non-affiliates, subjects needed to be able to distinguish callers based on their identity. To investigate individuality in female crested macaque alarm calls, we chose 20 females according to the strength of their social bond with the 10 females who were the subjects of the playback experiments. We obtained high-quality recordings of python-related alarm calls by presenting these females with an artificial python made of life-size picture mounted with a metal handle and presented in a realistic configuration (hereafter referred to as the model, see electronic supplementary material for an illustration).

The behaviour of the female and the calling bout were recorded with a high-definition camcorder (Panasonic, HDC-SD700) plugged-in with a directional microphone (Sennheiser K6-ME66, Wedemark, Germany, frequency response: 40–20 000 Hz ± 2.5 dB). During python model presentations, one experimenter, hidden behind a tree, slowly presented the model to the target female so that the head of the python would be visible above a root of the tree. A second experimenter recorded the behaviour of the target and the entire alarm calling bout. In an attempt to keep the perceived level of danger constant, the model was presented from a distance of 5–10 m to a female located at least 15 m away from the rest of the group. The python model was slowly moved out of sight of the female when she produced her first alarm call while the first experimenter remained hidden. This procedure allowed us to control for the distance separating the model from the target and for the exposure time to the stimulus, therefore minimizing the variation in the production of the alarm calls as well as maximizing the quality of the recordings. Presentations of the models were separated by at least 1 h (mean = 54.61 ± 48.35 h; range = 1.10–186.43 h), and we made sure that the group had moved to a new location, at least 150 m away from the location of the previous presentation. We carried out a maximum of two presentations per group and per day. We never presented the model twice to the same individual on the same day. All females reacted to the python model by uttering series of alarm calls and inspecting the area where the model was presented. In all cases, other individuals approached the caller and inspected the area, but none of them produced any alarm calls presumably because the model was then out of sight.

### (d) Acoustic analyses

We analysed the acoustic structure of 1171 single alarm calls given by 20 females from the two study groups. Calls stems from one alarm calling bout per female given in response to the presentation of the python model. Such bouts comprised up to 300 single calls and could last more than 5 min (mean = 190.03 ± 98.02 s). Individual calls were screened in SASLab Pro v. 5.1.20 (Avisoft Bioacoustics, Berlin, Germany) for background noises. Only high-quality calls were included in the analysis (mean = 58.55 ± 6.49; range: 24–92 calls per female). The calls were downsampled to 16 kHz and a 0.4 kHz high-pass filter was applied before measurements were carried out. Fast Fourier transformation (FFT) was then applied to appropriate calls (FFT length = 1024 points; window = Hamming; frame size = 100%; overlap = 96.87%) and the resulting spectrograms were then analysed in LMA v. 8.4 [45] (see electronic supplementary material).

A total of six parameters were measured. First, because calls were short (around 300 ms), and frequency modulation was mostly absent, we assessed the proportion of time segments in a given call that did not reveal a tonal structure. Given the predominantly noisy acoustic structure of calls, we used four parameters that describe very general patterns of energy distribution in the calls: (i) the median of the frequencies at which in each time segment the median value of the energy distribution was reached (DFA2), (ii) the first dominant frequency band (DFB1), (iii) the overall peak frequency (PF) and (iv) the frequency range (FR). Details and illustrations of these parameters can be found elsewhere [45–47]. As sixth parameter, we measured the duration of each call. The chosen spectral parameters have been previously used to describe primate vocalizations in general [47,48] and crested macaques' vocalization in particular [46].

### (e) Playback experiments

Assessment of the quality of the recording and preparation of the calls were carried out with SASLab Pro v. 5.1.20. The stimuli for the playback experiment were prepared by extracting a short series of several successive alarm calls from each recording. To maintain consistency between the stimuli, we selected only series of calls with high signal-to-noise ratio and matching temporal parameters (mean duration ± s.d. = 6.6 ± 0.2 s; mean calling rate ± s.d. = 1.5 ± 0.1 units s^−1^). Once edited, the calls were stored as ‘.wav’ files in a Marantz PMD660 Flash-Disc recorder (16 bits, PCM, frequency response: 20–20 000 Hz ± 3.0 dB, sampling rate: 44.1 kHz). The appropriate amplitude of the call series was judged in the forest, by a listener situated 15 m away from the speaker to match naturally occurring alarm calls. All calls were played back with the same amplitude.

We conducted 20 playback experiments on 10 adult females, following a within-subject design. On separate days, the same subject heard either a series of alarm calls produced by a closely associated female (‘affiliate’ condition; electronic supplementary material, video S2), or a series of alarm calls produced by an individual with whom the subject had a weak social bond (‘non-affiliate’ condition; electronic supplementary material, video S3). The order of the experiments was counterbalanced between the two conditions. Each call series was used only once as a stimulus. Alarm calls were played back from the recorder connected to a DavidActive speaker (Visonik, Germany, 30 W RMS, frequency response 120–20 000 Hz ± 1 dB) from a concealed location, 10–15 m away from the target individual. Before each trial, we ensured that the target had no visual access to the individual whose call was to be played and was sitting at least 20 m away from the group. Additionally, trials were carried out only if there had been no disturbances (predator encounters, intergroup encounters or alarm calls) during the 30 min preceding the experiment. To ensure that the subject reacted to the call being played back and not to events occurring in the group, and to facilitate the video-coding of the subject's reaction, the stimulus was played from a direction roughly perpendicular to the group's direction and between 45° and 135° to the target's body orientation (see illustration in the electronic supplementary material). Subjects were filmed for the entire duration of the stimulus and afterwards for 1 min. Successive playback experiments were separated by at least 2 h (mean: 66.56 ± 69.24 h; range: 2.33–218.80 h). We cannot exclude the possibility that other individuals than the target heard the stimulus (including targets of forthcoming experiments), but each subject was played back a unique series of alarm calls recorded from a different individual in each playback trial; a habituation effect is therefore unlikely.

### (f) Behavioural response

Frame-by-frame video analyses were used to examine the behaviour of the listener. In order to assess the possibility that the subject would show more willingness to engage in mobbing behaviour when hearing the call of a strongly bonded partner compared with a weakly bonded one, we measured: (i) the latency to react to the stimulus, defined as the time between the first call being played back and the first look towards the speaker; (ii) the duration of the orienting response, defined as the time spent looking towards plus the time approaching the speaker (to obtain a measure of the orienting response comparable across all range of situations; for example: looking only versus looking and approach) and (iii) the latency to approach the speaker, defined as the time between the first call and the first step towards the speaker. We also counted the number of approaches in each condition. We considered that the subject had oriented to or approached the speaker when the looking or walking direction was within roughly 22.5° of the speaker, at ground level (see illustration in the electronic supplementary material). The reaction of the target was coded for 30 s, following the offset of the stimulus. A naive observer coded half of the trials (*n* = 10) for reliability analyses. Inter-observer reliability tests showed a high level of agreement for the latency to react the stimulus (Spearman's rank correlation, *r* = 0.892, *p* = 0.001, *n* = 10), the duration of the orienting response (Spearman's rank correlation, *r* = 0.939, *p* < 0.001, *n* = 10), the number of approaches (Cohen's Kappa, *κ* = 1, *p* < 0.001, *n* = 10) and the latency to approach (Spearman's rank correlation, *r* = 0.986, *p* < 0.001, *n* = 10).

### (g) Statistical analyses

To test for individuality in female crested macaque alarm calls, we subjected the measured call parameters to linear discriminant function analysis. We analysed the calls separately for each social group with individual as grouping variable, thereby circumventing the problem of creating nested factors [49]. Because individuals contributed different numbers of calls, classification probabilities were adjusted accordingly. To validate the original classification results, we performed a cross-validation using the leave-one-out method that classifies each case based on functions derived by all but the one case [50]. Before running the analysis, we verified that all measurements meet the assumptions underlying the discriminant analysis procedure (see electronic supplementary material). Linear discriminant functions were derived using the R function ‘lda’ (package MASS, [51]).

For the playback experiments, we used the Wilcoxon signed-rank test to compare the duration of the overall orienting response, the latency to react and the latency to approach the speaker between the ‘affiliate’ and ‘non-affiliate’ conditions. Statistics were computed with R v. 15.0 [52]. All tests were exact, two-tailed with alpha set at 0.05. *p*-Value and effect sizes (d) are reported for each analysis.

## 3. Results

### (a) Individuality in female crested macaques alarm calls

All females reacted to the python model by uttering alarm calls and inspecting the area where it was presented. Each alarm calling bout (*n* = 20) elicited the approach of at least one conspecific, confirming the recruitment function of python-related alarm calls in this species. Discriminant function analyses revealed that in both groups, the calls were correctly classified above chance level, meaning that calls could be distinguished statistically on the basis of individual identity (table 1 and see electronic supplementary material for the descriptive statistics of the measured acoustic parameters).

**Table 1.** Results of the linear discriminant function analysis and classification results.

	R1 (*n* = 14)	PB (*n* = 6)
Wilk's lambda	0.1066	0.0901
*F*	25.704	45.618
d.f.	13 730	5419
*p*	<0.0001	<0.0001
classification		
total number of calls	744	425
expected classification	53 (7.1%)	71 (16.7%)
original classification	371 (49.9%)	280 (65.9%)
cross-validation	361 (48.5%)	274 (64.5%)

### (b) Playback experiments

**Figure 1.**
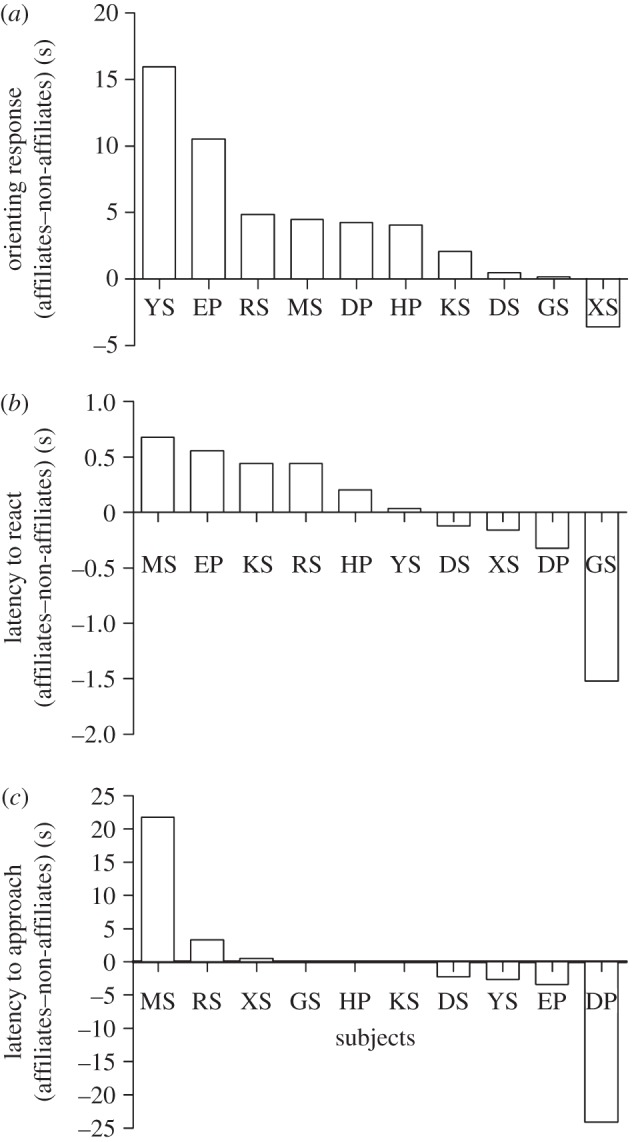
Females' responses to playbacks of close affiliates' and non-affiliates' alarm calls. The responses are expressed as the difference between subjects' response in the affiliate condition minus their response in the non-affiliate condition. (*a*) Overall orientation time towards the speaker: a positive difference indicates a longer orienting response in the affiliate condition compared with the non-affiliate condition. (*b*) Latency to react to the alarm calls being played back: a negative difference indicates a shorter latency to react to the alarm calls of a close affiliate. (*c*) Latency to approach the speaker: a negative difference indicates a shorter latency to approach the speaker when the calls of a close affiliate are being played back.

Frame-by-frame video analyses of the playback trials revealed that overall, subjects differed in their responses to alarm call series according to the strength of the social bond between caller and listener. All females looked towards the speaker when hearing the alarm call, and nine out of 10 individuals paid more attention to the calls of the affiliate. Analysis of the mean overall orienting responses showed that subjects' oriented to calls of affiliates 4.37 s (23%) longer than to calls of non-affiliates (Wilcoxon signed-rank test, Mdn_affiliates_ = 20.46 s, Mdn_non-affiliates_ = 13.66 s, *t* = 4, *p* = 0.014, *n* = 10, *d* = 0.536; figure 1*a*). The latency to react to the stimulus did not differ depending on the strength of the social bond between caller and listener (Wilcoxon signed-rank test, Mdn_affiliates_ = 0.3 s, Mdn_non-affiliates_ = 0.3 s, *t* = 20, *p* = 0.475, *n* = 10, *d* = 0.171) suggesting that all calls were equally salient and elicited extremely quick reactions (figure 1*b*). Six out of 10 individuals approached the speaker when hearing the call of an affiliate while five out of 10 approached when hearing a non-affiliate. The latency to approach the speaker did not differ between conditions (Wilcoxon signed-rank test, Mdn_affiliates_ = 16.9 s, Mdn_non-affiliates_ = 19.1 s, *t* = 11.5, *p* = 0.719, *n* = 10, *d* = 0.161; figure 1*c*).

**Figure 2.**
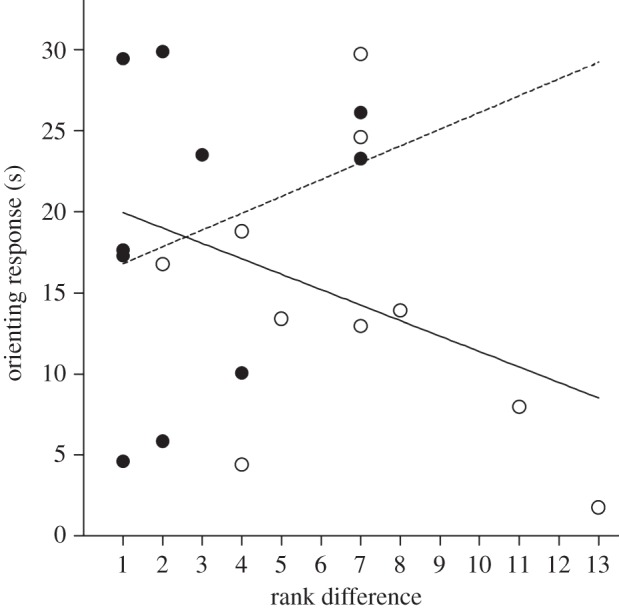
Correlations between the strength of subject females' responses and rank differences between callers and listeners. Subjects having a rank similar to the caller did not react more strongly than subjects having a big rank difference with the caller, regardless of the strength of the social bond between caller and listener. Filled circles, affiliates; open circles, non-affiliates.

Dominance and kinship can influence how individuals vocalize and respond to vocal signals in non-human primates [35,36,53]. Therefore, it could be that the strength of subjects' reaction depends on the rank of the caller rather than according to the strength of the social bond. This is unlikely, because we tested dyads with varying rank differences (from 1 to 13 ranks between the subject and the caller). In addition, females' responses did not correlate with rank difference (Spearman rank correlation, affiliates condition: *n* = 10, *r*_s_ = 0.214, *p* = 0.553; non-affiliates condition: *n* = 10, *r*_s_ = −0.339, *p* = 0.339; figure 2).

## 4. Discussion

Our results show that female crested macaques attend more to the alarm calls of affiliates compared with non-affiliates, highlighting the importance of social bonds in the life-threatening context of predator deterrence. Acoustic analysis of the alarm calls produced during the presentation of a predator model showed that the acoustic structure of adult female crested macaques' alarm calls contains sufficient information to allow listeners to identify the caller with a reasonable level of certainty, and to react accordingly. This result confirms previous findings on individually distinct alarm calls in a wide range of animals [22] and was a necessary prerequisite to assess the role played by social bonds in anti-predator behaviour.

Although individuals paid significantly more attention to the calls of affiliates, they did not react faster to these calls nor did they approach the speaker faster. The lack of difference between the two conditions could be due to the salience of alarm calls. As alarm calls are urgent signals, receivers should react quickly regardless of the caller, and then decide whether to keep looking towards the speaker or approach.

Seven out of 10 females approached the speaker, but they did not approach for alarm calls of close affiliates more often than those of more distant affiliates. Most likely, our stimulus did not induce approach in all individuals because it did not perfectly mimic the presence of a predator. Crested macaques' alarm calls are usually sustained for extended periods of time (up to 4.8 min), continuing even after the arrival of other individuals. Individuals joining the caller on the scene usually emit alarm calls themselves when sighting the predator. The number of callers usually increases over time, possibly increasing even more the number of individuals approaching. However, as we wanted to minimize the influence of the individuals surrounding the subject of the playback experiment, we needed a rather short stimulus. Furthermore, our main goal was to investigate the effect of the quality of the dyadic relationship between the caller and the listener therefore, we needed to simulate the presence of a single caller. Although this methodology was necessary to answer our question, it probably reduced the likelihood that the subject would approach the speaker. Nevertheless, individuals responded significantly more strongly to alarm calls produced by close affiliates compared with distant affiliates, confirming our prediction.

In crested macaques, python-related alarm calls function as recruitment alarm calls: they elicit the approach of other individuals who ultimately engage in mobbing behaviour, often leading the python to retreat. Mobbing poses risks onto the participants, and its success depends on coordination and cooperation [21,26]. Close affiliates are valuable social partners; individuals are more likely to receive support from close affiliates during aggressive interactions [12] and reconciliation rates are increased among them [7]. Competition is also reduced between close associates [54], and they are more prone to share resources, either actively or as a result of increased social tolerance [55,56]. Consequently, the costs of losing a prefered social partner might outweigh the costs of engaging in cooperative mobbing behaviour. This might especially be true considering the fact that the efficiency of mobbing behaviour increases when the number of individulals involved in mobbing increases, while the risk of being predated decreases when the number of mobbers increases [26].

One could argue that other factors than social bonds may have caused the observed response pattern. So far, differential response to alarm calls has been attributed to variation in the reliability of the caller [29], or scepticism from the listener [36]. By decreasing response to unreliable callers, listeners could optimize their anti-predator behaviour [30]. Because reliability was not manipulated in our study and because all recordings were all obtained in the same, highly controlled context where the predator was always present (i.e. model presentation), it seems unlikely that reliability of callers can explain our results. Reliability tends to be associated with age and social status: infants and juveniles frequently produce alarm calls in irrelevant situations [32], and high-ranking individuals are less likely to withhold information than low-ranking ones [35,36]. Our study focused only on adult females, thus ruling out an effect of age-related reliability, and the dominance relationship between callers and listeners had no effect on the behaviour of the listener. Moreover, encounters with pythons are frequent in crested macaques' habitat and predation has been observed several times, likely making deception a dangerous (or at least short-lived) strategy in this context. It is also worth mentioning that although alarm calls are often studied within the framework of active (i.e. providing false information) or passive deception (i.e. withholding information), evidence and systematic studies of such behaviour are rare [22].

In addition to caller reliability, the presence of kin in the audience has been shown to influence the production of and response to alarm calls during real or simulated encounters with predators [22]. In our study, the composition of the audience was controlled for, as model presentations and playback trials were systematically carried out with an isolated individual. Unfortunately, genetic relatedness is unknown for the two studied groups; so we cannot entirely rule out the possibility that dyads in the affiliate condition were genetically related. We thus took caution to reduce the probability of testing two related individuals. We used spatial distribution to characterize the strength of social bonds, knowing that in crested macaques, patterns of spatial distribution are not related to kinship networks [38]. Furthermore, in this species, kin bias is strikingly absent regarding the distribution of physical contact and grooming interactions [57], the second factor used in the calculation of the composite social index. In addition, it is well known for macaques that close kin share similar ranks [58]. We thus tested dyads with big rank differences. Finally, it has been shown for crested macaques that the strength of the social bond can be independent from kinship [19], a finding in agreement with the outcome of a number of other studies of crested macaque social behaviour, communication and cognition [38,59–62]. This combination of facts suggests that the probability for kinship to account for all the variance observed in our experiment is very low but we cannot rule out this possibility.

Previous studies also showed that variation of the acoustic structure of alarm calls related to arousal of the caller and perceived urgency of the threat impacts on the production and perception of the calls [22]. However, the rigorous design of the experiment allows us to discard these explanations for the increased response towards close affiliates in our study. Indeed, both the model presentation and the playback trials were highly standardized: in each case, the stimulus was presented from the same distance for all targets, and for similar duration. While the acoustic analyses revealed individual differences in the structure of the alarm calls, these cannot be attributed to variation in arousal of perceived urgency but only as individual characteristics.

Direct comparison of our results with those obtained in previous studies is difficult because of methodological differences, and because it is the first time that the role of social bonds is investigated in this context. However, some evidence converges to suggest that responses to anti-predator alarm calls could be influenced by species social style. Crested macaques are considered as more socially tolerant than other macaques species: dominance and kinship usually have little influence on their social behaviour compared with affiliation [19,37,38], which corresponds to the pattern of response observed in our study (i.e. no effect of dominance relationships, but strong effect of social bonds). Interestingly, in the less tolerant rhesus macaque, social status seems to play a highly influencial role in alarm calling behaviour [36]. Such co-variation between the factors affecting responses to alarm calls and social tolerance would support previous findings highlighting the central importance of dominance and kinship on the social behaviour, communication and cognition of less tolerant species, whereas in more tolerant species, social bonds seem to have more weight [19,38,62–65]. More work is needed to better understand the influence of species' social style on the factors affecting cooperative defence, as well as the selective forces driving its evolution. In particular, the influence of strong social bonds on anti-predator behaviour needs to be investigated in less tolerant macaque species.

Overall, our results demonstrate that the strength of social bonds is a factor that individuals are sensitive to when attending to anti-predator recruitment alarm calls. Combined with previous studies, our results contribute to the growing evidence showing that strong positive bonds, just like dominance or kinship, are a crucial feature of animal societies [4,6], and that they can provide individuals with advantages beyond the within-group social context. Predation pressures undeniably played a role in the evolution of sociality [26]; however, new costs arose with the formation of large social groups and complex social relationships, and evidences converge to suggest that the formation of close social bonds can be a powerful way to mitigate these costs, whether these are increased competition [24], stress [66], infanticide [15] or protection against predators.
